# Maternal early-pregnancy body mass index-associated metabolomic component and mental and behavioral disorders in children

**DOI:** 10.1038/s41380-022-01723-3

**Published:** 2022-08-10

**Authors:** Polina Girchenko, Marius Lahti-Pulkkinen, Jari Lipsanen, Kati Heinonen, Jari Lahti, Ville Rantalainen, Esa Hämäläinen, Hannele Laivuori, Pia M. Villa, Eero Kajantie, Katri Räikkönen

**Affiliations:** 1 Department of Psychology and Logopedics, Faculty of Medicine, University of Helsinki, Helsinki, Finland; 2 National Institute for Health and Welfare, Helsinki, Finland; 3 Queen’s Medical Research Institute, University of Edinburgh, Edinburgh, UK; 4 Psychology/ Welfare Sciences, Faculty of Social Sciences, Tampere University, Tampere, Finland; 5 Department of Clinical Chemistry, University of Eastern Finland, Kuopio, Finland; 6 Department of Obstetrics and Gynecology, Tampere University Hospital, Tampere, Finland; 7 Center for Child, Adolescent and Maternal Health Research, Faculty of Medicine and Health Technology, Tampere University, Tampere, Finland; 8 Medical and Clinical Genetics, University of Helsinki and Helsinki University Hospital, Helsinki, Finland; 9 Institute for Molecular Medicine Finland, Helsinki Institute of Life Science, University of Helsinki, Helsinki, Finland; 10 Obstetrics and Gynaecology, Helsinki University Hospital and University of Helsinki, Helsinki, Finland; 11 National Institute for Health and Welfare, Public Health Promotion Unit, Helsinki, Finland; 12 University of Oulu, Oulu, Finland

**Keywords:** Predictive markers, Psychiatric disorders

## Abstract

Maternal pre-pregnancy obesity and/or higher body mass index (BMI) have been associated with neurodevelopmental and mental health adversities in children. While maternal metabolomic perturbations during pregnancy may underpin these associations, the existing evidence is limited to studying individual metabolites, not capturing metabolic variation specific to maternal BMI, and not accounting for the correlated nature of the metabolomic measures. By using multivariate supervised analytical methods, we first identified maternal early-pregnancy BMI-associated metabolomic component during pregnancy. We then examined whether this component was associated with mental and behavioral disorders in children, improved the prediction of the child outcomes over maternal BMI, and what proportion of the effect of maternal BMI on the child outcomes this component mediated. Early-pregnancy BMI of 425 mothers participating in the PREDO study was extracted from the national Medical Birth Register. During pregnancy, mothers donated up to three blood samples, from which a targeted panel of 68 metabolites were measured. Mental and behavioral disorders in children followed-up from birth until 8.4–12.8 years came from the Care Register for Health Care. Of the 68 metabolites averaged across the three sampling points, 43 associated significantly with maternal early-pregnancy BMI yielding a maternal early-pregnancy BMI-associated metabolomic component (total variance explained, 55.4%; predictive ability, 52.0%). This metabolomic component was significantly associated with higher hazard of any mental and behavioral disorder [HR 1.45, 95%CI(1.15, 1.84)] and relative risk of having a higher number of co-morbid disorders [RR 1.43, 95%CI(1.12, 1.69)] in children. It improved the goodness-of-model-fit over maternal BMI by 37.7–65.6%, and hence the predictive significance of the model, and mediated 60.8–75.8% of the effect of maternal BMI on the child outcomes. Maternal BMI-related metabolomic perturbations during pregnancy are associated with a higher risk of mental and behavioral disorders in children. These findings may allow identifying metabolomic targets for personalized interventions.

## Introduction

Maternal pre-pregnancy obesity (Body Mass Index [BMI] ≥ 30 kg/m^2^) has become a major challenge of obstetric care. In 2016, the global prevalence of obesity in women aged 18 years and older was 15% [1] and by 2025 it is projected to exceed 21% [2]. In the mother, pre-pregnancy obesity increases the risk of gestational diabetes, hypertensive disorders, caesarian section and preterm delivery and is often co-morbid with mental health problems, including depressive symptoms [3–6]. In the children, maternal pre-pregnancy obesity increases the risk of both intrauterine growth restriction (IUGR) and macrosomia, as well as congenital anomalies and fetal death [6, 7]. Mounting evidence suggests that the risks in the children also extend to neurodevelopmental and mental health adversities later in life [8–12].

The risks on neurodevelopmental and mental health adversities in the children have been suggested to be, at least in part, explained by obesity-related perturbations in maternal metabolome and in maternal-fetal metabolomic communication [13]. By using targeted metabolomic profiling and untargeted metabolomic fingerprinting, previous studies have demonstrated that maternal pre-pregnancy obesity and/or higher BMI are associated with perturbations in many metabolic pathways, including lipids [13–17], amino acids (AA) [13–15, 17], fatty acids (FA) [14–17], glycolysis [14, 15], ketone bodies [14–16], and inflammation [14, 15, 17] during pregnancy, exceeding the perturbations that are induced by the pregnancy in itself. We have recently contributed to this literature by showing that in overweight and obese pregnant women metabolites across the above-mentioned pathways are perturbed throughout pregnancy, and pregnancy-induced changes in these metabolites are smaller [14], implying that overweight and obese pregnancies are characterized by metabolic inflexibility.

To date, we are not aware of studies that would have examined maternal obesity- or BMI-associated metabolomic profiles during pregnancy as predictors of child neurodevelopmental or mental health outcomes. The existing targeted metabolomics studies have focused on individual metabolites in the pathways related to polyunsaturated fatty acids (PUFA) and inflammation, and have focused on autism spectrum disorders (ASD) or traits and neurodevelopmental delay [18–20]. Similarly, while the untargeted metabolomic studies have covered a much larger number of metabolic pathways, they are also limited to studying individual metabolites and ASD or non-typical neurodevelopment [21, 22].

Moreover, none of the existing studies have taken advantage of multivariate supervised analytical methods that have become the mainstream of metabolomics analyses, such as orthogonal partial least squares (O-PLS) analysis. Hence, variation in the individual metabolites of the mother during pregnancy examined in the previous studies [18–22] may not have necessarily reflected the metabolomic variation specific to maternal obesity or higher BMI during pregnancy, and has not accounted for the correlated nature of the metabolomic measures. O-PLS overcomes these limitations as it reduces data dimensions and finds latent variables that maximize the correlation between the predictors and the outcome, while taking advantage of an orthogonal signal correction filter to remove variation in the predictors that are not explained by the outcome [23]. As a result, O-PLS allows to cluster predictive information into one predictive component and simplify interpretability due to the decreasing of confounding effects stored in model components. Hence, predictive component derived from the O-PLS represents variation in the predictors specifically explaining variation in the outcome, and variation in the predictors extracted from the O-PLS can be subjected to further analyses [24]. In application to metabolomics, predictive component extracted from the O-PLS represents the metabolomic profile specific, for instance, to maternal BMI.

By applying the O-PLS analytic approach, we first identified maternal early-pregnancy BMI-associated metabolomic component during pregnancy; this metabolomic component represented maternal early-pregnancy BMI-related metabolomic profile. We then extracted this early-pregnancy BMI-associated metabolomic component and examined whether it predicted mental and behavioral disorders in the children followed-up from birth until 8.4–12.8 years. Next, we tested if this metabolomic component improved the goodness-of-model-fit over maternal early-pregnancy BMI, and hence improved the predictive significance of the model in identifying the risk of mental and behavioral disorders in children. Finally, we estimated the effect size proportion mediated by this metabolomic component of the effect of maternal early-pregnancy BMI on mental and behavioral disorders in children. We used targeted high-throughput proton nuclear magnetic resonance (NMR)-based metabolomics platform, which yielded metabolites covering pathways related to lipids, FAs, AAs, ketone bodies, fluid balance, glycolysis and inflammation.

## Materials and methods

### Participants

Study participants come from the Prediction and Prevention of Preeclampsia and Intrauterine Growth Restriction (PREDO) study [25]. In 2005–2009, we enrolled 1079 pregnant mothers to the clinical study sample: 969 had one or more and 110 had none of the known risk factors for preeclampsia and intrauterine growth restriction. The women were recruited when they arrived for their first ultrasound screening at 12–14 gestational weeks conducted at 10 study hospitals in Southern and Eastern Finland.

Of the 1079 pregnant mothers, 425 donated blood at up to three times during pregnancy. Economic constraints restricted blood sampling to the three largest study hospitals. Blood samples were taken at median 13.0 (interquartile range (IQR) 12.6–13.4), 19.3 (IQR 19.0–19.7), and 27.0 (IQR 26.6–27.6) gestational weeks. Of the 425 mothers, 354 (83.8%) provided blood samples at all three time points, 52 (14.6%) at two time points, and 10 (2.4%) at one time point. All 425 children of these women (0% data attrition), had data on mental and behavioral disorder diagnoses in a follow-up from birth until the child’s age of 8.4–12.8 years.

In comparison to the mothers who did not donate blood, mothers in the subsample who did were younger (32.5 vs. 33.6 years; *p* = 0.001) and less likely to be obese (29.2% vs. 39.6%; *p* = 0.005), and their children were followed-up for a longer period of time (10.4 vs. 10.1 years; *p* < 0.0001), but there were no significant differences in the other study variables (*p* > 0.07).

The PREDO study protocol was approved by ethics committees of the Helsinki and Uusimaa Hospital District and aligns with the Code of Ethics of the World Medical Association (Declaration of Helsinki). All participants provided written informed consent. Consent for participating children was provided by parent(s)/legal guardian(s). The consent enabled linkage to nationwide medical register data for the women and the children using unique personal identification numbers assigned to all Finnish citizens and residents since 1971.

### Maternal early-pregnancy BMI

Data were extracted from the Medical Birth Register (MBR). Early-pregnancy BMI was calculated from weight and height verified at the first visit to the antenatal clinic at a mean of 8 + 4 weeks + days of gestation (SD  =  1  +  3 weeks + days of gestation).

### Maternal metabolomic profiling during pregnancy

Venous blood samples were drawn from the antecubital vein between 7 and 10 AM after at least a 10-h overnight fast. Plasma was separated immediately and stored at −80 °C until analysis, in which 225 metabolites were quantified by using a high-throughput proton NMR metabolomics platform (Nightingale Health Ltd, Helsinki, Finland). The metabolites included 186 lipoprotein lipids and their subclasses, 9 FAs and 7 ratios of FAs, 5 other lipids, 8 AAs, 3 ketone bodies, 2 metabolites related to fluid balance, 3 to glycolysis and 1 to inflammation. Following the lead of earlier studies using this metabolomics platform, we used 68 of these metabolic measures as our primary variables [14, 15, 26]. However, we show the results also for the entire panel of metabolites. Details of the experimentation and applications of the NMR metabolomics platform have been described previously [27]. This NMR platform has been used in studies of pregnant populations [14, 15, 26]. Of all the metabolites, 37 have been validated against standard clinical chemistry methods.

All metabolite concentrations were log-transformed and standardized to the mean of 0 and standard deviation (SD) of 1 across all three time points. All values above and below 5 SDs from the mean were considered outliers and recoded as missing values. Because the metabolites showed high intra-class correlations across the three consecutive measurement points during pregnancy (*R* = 0.56–0.89, *p* < 0.001) and as our previous analysis showed that obese pregnancies were characterized by persistent metabolic perturbations throughout pregnancy and smaller change across the three measurement points [14], we used their average in the analyses.

### Mental and behavioral disorders in children

We identified mental and behavioral disorder diagnoses in children from the Care Register for Health Care (HILMO) from birth until 31/12/2018 when the children were 8.4–12.8 years of age. Our primary outcomes were any mental or behavioral disorder diagnosis (categorized as yes vs. no) (International Statistical Classification of Diseases and Related Health Problems, tenth revision [ICD-10] codes: F00−F99) and their co-morbidity (categorized as zero, one, two or more). HILMO has high validity for mental and behavioral disorder diagnoses [28, 29].

### Covariates

Covariates derived from the MBR and/or HILMO included maternal age in early pregnancy (<40/≥ years), parity (primiparous/multiparous), child’s sex and birth year. Maternal education level (secondary or lower/tertiary) was self-reported in early pregnancy. Maternal substance use during pregnancy comprised MBR data on smoking (no smoking/quit during 1st trimester or smoked throughout pregnancy) and self-reported data on alcohol consumption (no/yes) during early pregnancy.

### Statistical analyses

We examined multivariate associations between maternal early-pregnancy BMI and maternal metabolites during pregnancy by using O-PLS regression. The model fit was evaluated by the total amount of variation explained (R2) and by the predictive ability of the model as determined by a sevenfold cross-validation (Q2). We derived maternal early-pregnancy BMI-associated metabolomic component from the O-PLS model. This metabolomic component represented maternal metabolomic profile during pregnancy specific to her early-pregnancy BMI. By using Cox proportional hazards models, we then examined if this maternal BMI-associated metabolomic component was associated with mental and behavioral disorders in children. The children were followed-up from birth until the first mental and behavioral disorder diagnosis and were censored at death, emigration or December 31 2018; in this sub-sample none of the children died or moved abroad during the follow-up. Cox models are ideal in cases where the follow-up time varies by individual. Before applying Cox models, we assured that there were no time-dependent effects. We used Poisson regression analyses to examine if the maternal BMI-associated metabolomic component was associated with co-morbidity of mental and behavioral disorders in children.

To examine whether maternal BMI-associated metabolomic component improved the prediction of the risk mental and behavioral disorders in children over maternal BMI, we tested the goodness-of-fit of two nested models by using the likelihood ratio chi-square test (LRT). We compared the fit of a baseline model, in which maternal early-pregnancy BMI was the sole predictor of mental and behavioral disorders in children, with the fit of a model, which included both the maternal early-pregnancy BMI and the maternal metabolomic component predictive of BMI as the predictors.

To examine the effect size proportion mediated by the maternal BMI-associated metabolomic component of the effect of maternal early-pregnancy BMI on child mental and behavioral disorders, we used structural equation modeling (SEM) with 10,000 bootstrap replications [30] when we used any mental and behavioral disorder diagnosis as an outcome, and Sobel test [31] when we used co-morbidity of mental and behavioral disorders as an outcome. We proceeded to the mediation analyses in case the criteria for mediation were met, namely that the model fit of the O-PLS was acceptable and yielded a meaningful maternal metabolomic component predictive of BMI, and that maternal early-pregnancy BMI and the BMI-associated metabolomic component were associated with mental and behavioral disorders in children.

We present the associations adjusted for child’s sex and birth year (Model 1), and then for all covariates (Model 2). As effect sizes we present Hazard Ratios (HR) and Risk Ratios (RR) with 95% confidence intervals (CI), LR chi-square values, and the proportion of the total effect that the maternal BMI-associated metabolomic component mediated of the effect of maternal early-pregnancy BMI on mental and behavioral disorders in children. As supplementary analyses, we show multivariate associations between maternal early-pregnancy BMI and the full metabolomics panel comprising 225 metabolic measures, and the associations of this maternal metabolomic component predictive of BMI with mental and behavioral disorders in children.

O-PLS regression analyses were performed using SIMCA (Version 17.0, Umetrics, Sweden). Cox proportional hazards and Poisson regression were performed using SAS 9.4 (SAS Institute Inc., Cary, NC, USA) and mediation analysis was performed using Stata 15 (StataCorp. 2017. Stata Statistical Software: Release 15. College Station, TX, USA: StataCorp LLC).

## Results

Descriptive characteristics of the sample with data on maternal pregnancy metabolomics are shown in Table 1. Of the mothers 20.9% (*n* = 89) were overweight (BMI > 25 kg/m^2^) and 29.2% (*n* = 132) were obese. Of the children 12.9% (*n* = 55) had any mental and behavioral disorder diagnosis; 5.9% (*n* = 25) had one and 7.1% (*n* = 30) had two or more different mental and behavioral disorder diagnoses.

**Table 1:** Characteristics of the study population (*n* = 425 mother-child dyads).

Maternal characteristics:	Mean (SD) or *N* (%)
Maternal age in early pregnancy, years	32.5 (5.3)
Maternal age younger than 40 years	382 (90.9%)
Maternal age 40 years and older	43 (10.1%)
Data not available	0
Education level	
Upper secondary or less	204 (49.0%)
Tertiary	212 (51.0%)
Data not available	9 (2.1%)
Parity	
Primiparous	137 (32.2%)
Multiparous	288 (67.8%)
Data not available	0
Smoking or alcohol use at any point during pregnancy	
No	349 (82.1%)
Yes	76 (18.9%)
Data not available	0
Early-pregnancy body mass index kg/m^2^	27.0 (6.5)
Overweight (25–30 kg/m^2^)	89 (20.9%)
Obese (≥30 kg/m^2^)	124 (29.2%)
Data not available	0
**Children’s characteristics:**	
Sex	
Boy	228 (53.7%)
Girl	197 (46.4%)
Data not available	0
Follow-up length years (Median, IRQ)	10.4 (9.4–11.3)
Data not available	0
Any mental and behavioral disorder diagnosis	
No	370 (87.1%)
Yes	55 (12.9%)
Data not available	0
Co-morbidity of mental and behavioral disorder diagnoses	
Zero	370 (87.1%)
One	25 (5.9%)
Two and more	30 (7.1%)
Data not available	0

### Maternal early-pregnancy BMI-associated metabolomic component

**Fig. 1:**
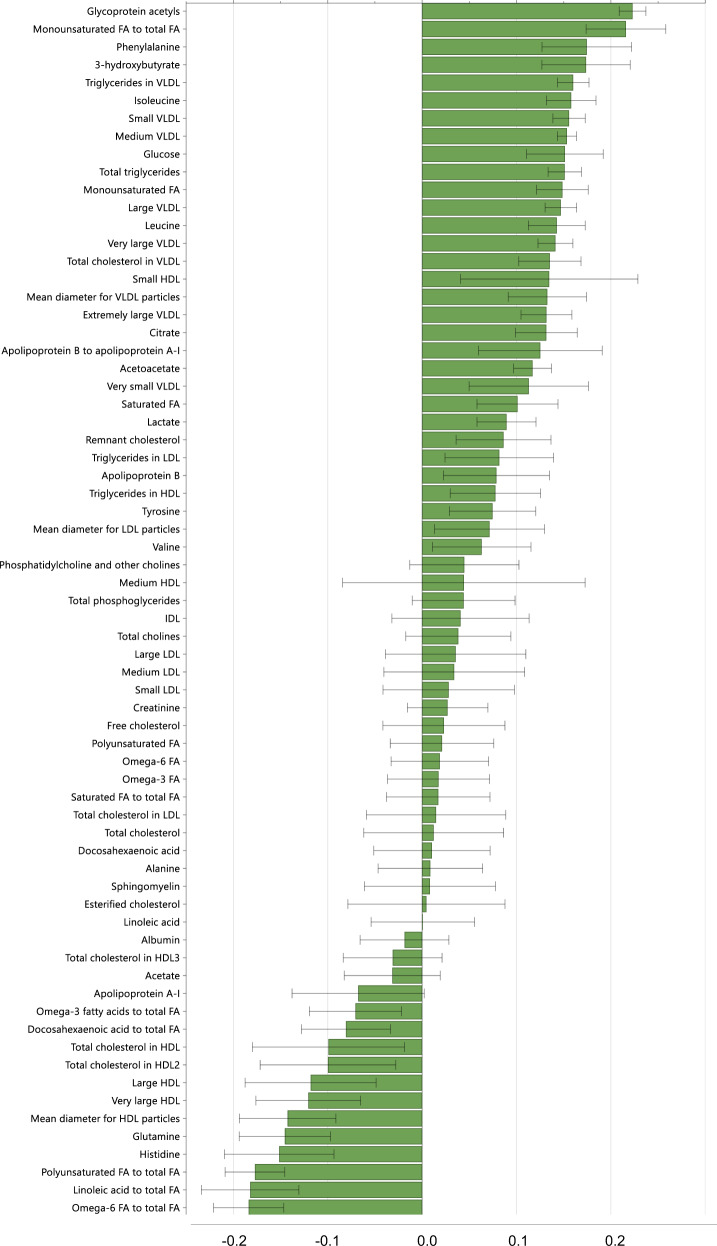
Loadings of the 68 metabolites on the maternal early-pregnancy BMI-associated metabolomic component.

O-PLS testing associations between maternal early-pregnancy BMI and the 68 maternal metabolites during pregnancy yielded a model, which explained 55.4% of the total variation of maternal early-pregnancy BMI (R2) and had a predictive ability (Q2) of 52.0%. Loadings of the 68 metabolites on maternal BMI-associated metabolomic component are shown in the Fig. 1. Of the 68 metabolites, 43 contributed to explaining variation in maternal early-pregnancy BMI. Major contributing metabolites with the highest positive loadings on the metabolomic component were glycoprotein acetyls, monounsaturated FA to total FA ratio, phenylalanine, 3-hydroxybuyrate, glucose and VLDL-, remnant cholesterol-, and apolipoprotein-related metabolites, and with highest negative loadings were ratios of ω-6, linoleic acid and PUFA to total FA, histidine, glutamine and HDL-related metabolites.

### Maternal early-pregnancy BMI-associated metabolomic component and mental and behavioral disorders in children

**Fig. 2:**
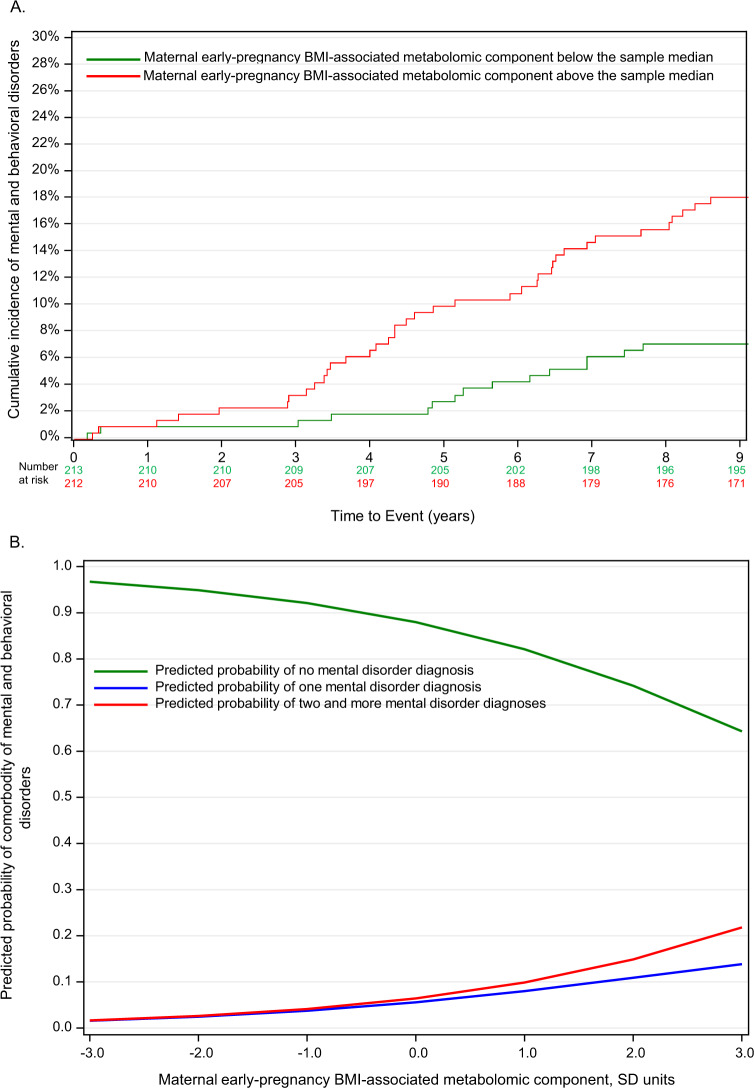
Maternal BMI-associated metabolomic component and mental and behavioral disorders in children. **A** Cumulative incidence of mental and behavioral disorders in the children of the mothers with the maternal early-pregnancy body mass index (BMI)-associated metabolomic component during pregnancy below and above the median; **B** Probability of the children to have a higher number of co-morbid mental and behavioral disorder diagnoses according to each standard deviation unit increase in the level of the maternal early-pregnancy BMI-associated metabolomic component.

Table 2 shows that maternal BMI-associated metabolomic component was significantly associated with higher hazard of any mental and behavioral disorder and higher RR of having a higher number of co-morbid disorders in children. These associations were not explained by the covariates. Figure 2 displays these associations graphically.

**Table 2:** Associations between maternal early-pregnancy body mass index (BMI)-associated metabolomic component and maternal early pregnancy BMI and mental and behavioral disorders in children.

Outcomes in children						
Maternal predictors	Any mental and behavioral disorder diagnosis (no vs yes)			Co-morbidity of mental and behavioral disorder diagnosis (zero, one, two or more diagnoses)		
	HR	95% CI	p	RR	95% CI	p
Maternal early-pregnancy BMI-associated metabolomic component (SD units)						
Model 1	1.49	1.18, 1.88	0.0009	1.43	1.18, 1.73	0.0002
Model 2	1.41	1.09, 1.82	0.009	1.37	1.12, 1.68	0.003
Maternal early-pregnancy BMI						
(SD units)						
Model 1	1.35	1.07, 1.70	0.01	1.34	1.11, 1,62	0.002
Model 2	1.26	0.98, 1.61	0.11	1.27	1.03, 1.55	0.02

HR refers to Hazard Ratio of any mental and behavioral disorder diagnosis, RR to Relative Risk of having a higher number of mental and behavioral disorder diagnosis, and 95% CI to 95% Confidence Interval.

Model 1 is adjusted for child’s sex and birth year; Model 2 is adjusted for Model 1 covariates and maternal age, education and substance-use during pregnancy.

### Comparison of goodness-of-fit of the two nested models

The LR chi-square values of the baseline models, in which maternal early-pregnancy BMI was the sole predictor of any mental and behavioral disorder and co-morbidity of mental and behavioral disorders in children, were 6.7 (*p* < 0.001) and 10.6 (*p* < 0.001) respectively. In these models, maternal early-pregnancy BMI was a significant predictor of higher hazard of any mental and behavioral disorder and higher RR of having a higher number of co-morbid disorders in children (Table 2). The association with any mental and behavioral disorder was rendered non-significant in a model adjusting for all covariates, while the association with the co-morbidity of disorders remained significant (Table 2). Addition of the maternal BMI-associated metabolomic component into the baseline models further improved the goodness-of-fit: LR chi-square values for the metabolomic component were 4.4 (*p* = 0.04) and 4.0 (*p* = 0.05), respectively. The model fit showed 65.7% improvement in prediction of any mental and behavioral disorder and 37.7% improvement in prediction of co-morbidity of mental and behavioral disorders. In the model with both the maternal early-pregnancy BMI and the maternal BMI-associated metabolomic component as predictors of child outcomes, maternal early-pregnancy BMI was no longer a significant predictor of any mental and behavioral disorder (HR 1.03,95%CI 0.73, 1.46, *p* = 0.85) or the co-morbidity of mental and behavioral disorders (RR 1.09,(95%CI 0.82, 1.45, *p* = 0.56)), while the metabolomic component was (HR 1.47, 95% CI 1.04, 2.08, *p* < 0.03; RR 1.37, 95% CI 1.03, 1.82, *p* < 0.03).

### The effect size proportion mediated by maternal early-pregnancy BMI-associated metabolomic component

**Fig. 3:**
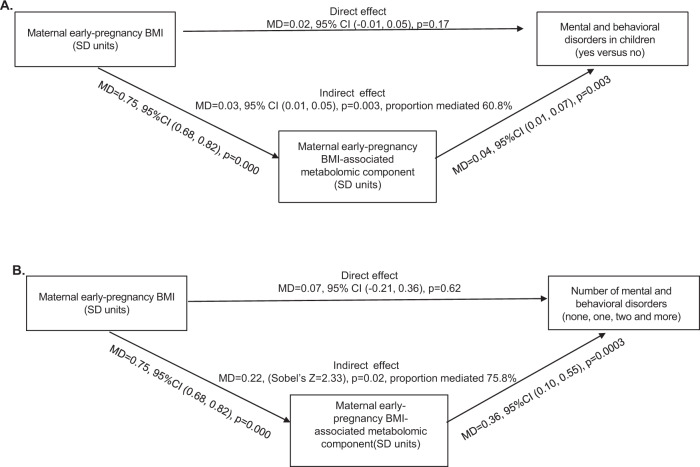
Mediation analysis. **A** Maternal early-pregnancy BMI-associated metabolomic component showing mediation of 60.8% of the effect of maternal early-pregnancy BMI on any mental and behavioral disorder. **B** Maternal early-pregnancy BMI-associated metabolomic component showing mediation of 75.8% of the effect of maternal early-pregnancy BMI on co-morbidity of mental and behavioral disorders in children.

Figure 3 shows that the proportion mediated by maternal early-pregnancy BMI-associated metabolomic component of the total effect of maternal early-pregnancy BMI on any mental and behavioral disorder in children was 60.8% (Panel A) and 75.8% on co-morbidity of mental and behavioral disorders (Panel B). Mediation analyses were conducted with the child’s sex and birth year as covariates. In the mediation models, the direct effect of maternal early pregnancy BMI on mental and behavioral disorder in children was no longer significant (Fig. 3).

### Supplementary analyses

Supplemental Table 1 shows the loadings of the metabolites on the BMI-associated metabolomic component derived from the O-PLS using the entire panel of 225 maternal metabolites during pregnancy. The total amount of variation (R2) in maternal early-pregnancy BMI explained by 225 metabolites was 58.4% and predictive ability of the model (Q2) was 53.9%. The 3% increase in the amount of the variation explained and the 1.9% increase in predictive ability by the 225 metabolites compared with the 68 metabolites suggested that the 68 metabolites covered well the maternal BMI-associated metabolomic variation during pregnancy. Associations between this maternal metabolomic component with mental and behavioral disorders in children were similar to the associations of metabolomic component based on the 68 maternal metabolites during pregnancy (Supplemental Table 2).

## Discussion

This study showed that of the 68 metabolites used as the primary metabolomic measures, 43 clustered significantly with maternal early-pregnancy BMI yielding a metabolomic component reflecting maternal metabolomic BMI-related profile during pregnancy. This BMI-associated metabolomic component was significantly associated with higher hazard of any mental and behavioral disorder and with a higher RR of having a higher number of co-morbid disorders in children. Comparison of the two nested models showed that a model, which comprised maternal early-pregnancy BMI and BMI-associated metabolomic component as predictors of mental and behavioral disorders in children, was superior in prediction of mental and behavioral disorders in children by 47.7–67.7% over a model in which maternal early pregnancy BMI was the sole predictor. Moreover, this study showed that over 60% of the total effect of maternal BMI on the mental health outcomes in children was mediated by this BMI-associated metabolomic component.

Our findings are in agreement with the results of previous pregnancy metabolomics studies of maternal BMI. These studies have, however, examined associations between maternal BMI during pregnancy and individual metabolites in the metabolic pathways related to lipids [13–17], AA [13–15, 17], FA [14–17], glycolysis [14, 15], ketone bodies [14–16] and inflammation [14, 15, 17]. Our findings are also in agreement with the few existing studies highlighting associations between maternal PUFAs and inflammation in relation to child ASD and related-traits [18–20]. By taking advantage of the O-PLS analytic approach, we analyzed the metabolites in all of these pathways simultaneously, while taking into account the correlated nature of these metabolomic measures. This approach reduced confounding and increased specificity of the identified metabolomic profile to maternal early-pregnancy BMI. Moreover, this approach allowed us to unravel the relative contributions of the individual metabolites on the BMI-associated metabolomic component. Variation in metabolites altogether explained 55.4% of the total variation in maternal early-pregnancy BMI and had 52% predictive significance. The 43 metabolites that clustered significantly onto the metabolomic BMI-associated component included higher levels of metabolites in the pathways related to inflammation, monounsaturated FA, aromatic AA, ketone bodies, glycolysis and VLDL-, remnant cholesterol- and apolipoprotein-lipids, and lower levels of ω-6 and linoleic FAs and PUFA to total FAs, and HDL-lipids.

Also, in accordance with the results of previously published studies [8], in our sample higher maternal early-pregnancy BMI was associated with a higher hazard of any mental and behavioral disorder in children and with a higher RR of having a higher number of co-morbid mental and behavioral disorder diagnoses. However, a model which also comprised maternal early-pregnancy BMI-associated metabolomic component as a predictor of mental and behavioral disorders in children was superior in prediction of mental and behavioral disorders in children over a model comprising maternal early-pregnancy BMI alone. In all adjustment models, maternal BMI-associated metabolomic component was associated with the higher hazard of any mental and behavioral disorder and a higher RR to have two or more diagnosed disorders in children, while the associations of maternal early-pregnancy BMI with mental and behavioral disorders were not significant in the models, which included the BMI-associated metabolomic component. These findings, thus, suggest that maternal metabolomic dysregulation during pregnancy may be among the biological mechanisms that plays a role in underlying the association between maternal early-pregnancy BMI and the risk of mental and behavioral disorders in children.

Mediation analyses further corroborated this hypothesis. The proportion of the effect of maternal early-pregnancy BMI on mental and behavioral disorders and their co-morbidity in children mediated by maternal early-pregnancy BMI-associated metabolomic component was over 60%. While the effect size proportion mediated was substantial, mediation was not full, suggesting that other biological, psychological or social factors may also play a role as mediators of this association.

Indeed, both maternal BMI and the risk of mental and behavioral disorders in children have been linked to many other factors, including socioeconomic disadvantage and poor lifestyle [32], and these factors have been shown to affect the associations between maternal BMI and child mental health outcomes [33]. In agreement with the existing evidence, when we made adjustments for maternal education, alcohol consumption and smoking during pregnancy, the association of maternal early-pregnancy BMI with any mental and behavioral disorder in the children was rendered non-significant. At the same time, all associations between maternal BMI-associated metabolomic component and mental and behavioral disorders in children remained significant across all models. These findings suggest that application of the O-PLS method allowed us to reduce confounding by the factors related to BMI and disentangle metabolic perturbations from the other factors related specifically to BMI.

Maternal metabolomic perturbations associated with higher maternal early-pregnancy BMI can affect fetal development via alterations in placental development [34–37] or directly through altering fetal exposure to an adverse maternal metabolomic milieu: increased or decreased transfer of metabolites to the fetus can lead to suboptimal composition of fetal nutrient supply where fetal demand and maternal supply are in mismatch [13]. Maternal metabolomic perturbations linked in our study with the maternal higher early-pregnancy BMI have been associated with oxidative stress, inflammation-induced mal-programming, dysregulation of insulin, glucose, and leptin signaling in the developing brain [38–40]. All of these mechanisms have been proposed to underlie the risk of neurodevelopmental adversities in the children of pregnant women with obesity or higher BMI [20, 41–43]. Hence, metabolic perturbations associated with higher maternal BMI during pregnancy may trigger alterations in maternal-fetal communication at many biological levels.

Our findings call for interventions that target metabolomic perturbations in the mothers with higher BMI during pregnancy. However, previous intervention studies promoting maternal physical activity, healthy dietary intake or micronutrient supply during pregnancy have resulted in a mixed pattern of findings. For instance, one intervention study, which targeted maternal dietary intake during pregnancy, showed benefits for maternal cholesterol levels, but maternal triglyceride levels remained unaffected [44]. One other intervention study targeting maternal dietary intake and physical activity during pregnancy showed benefits for maternal phospholipids and triglycerides in extremely large, very large, large and medium VLDL particles, mixed pattern or modest benefits for maternal FA ratios, whereas maternal total triglycerides and total cholesterol levels remained unaffected [45]. Yet another intervention study targeting diet and exercise during pregnancy did not result in any metabolic changes [14]. Furthermore, two other intervention studies targeting maternal omega-3 FA supply during pregnancy showed benefits for maternal triglyceride levels [46] and inflammation [47], while maternal cholesterol levels [46], fasting plasma glucose [47] and lipid levels [47] remained unaffected. These intervention studies demonstrate that while the metabolomic measures are correlated, the interventions do not necessarily associate with benefits across all the measured metabolomic pathways. Our finding highlighting that in the mothers with higher BMI the risk to the child neurodevelopment is based on dysregulation in 43 metabolites suggests that the interventions may need to be multimodal, targeting these specific metabolomic perturbations. Based on our findings, such multimodal interventions would be expected to carry benefits on child mental health outcomes, however, future studies are needed to confirm this hypothesis.

Study strengths include the prospective study design, well-characterized sample, a large, targeted set of metabolites measured throughout pregnancy and from blood samples taken in the morning after a 10-hour fast, and data extracted from nationwide medical registers resulting in null data attrition in the child follow-up. Our study limitation is that 90% of the mothers were recruited into the study based on their risk factor status for preeclampsia and IUGR, which resulted in overrepresentation of obesity and cardiometabolic pregnancy conditions in our sample. This study limitation should, however, be evaluated in the light of baseline enrollment, which for the eldest mothers and children occurred 16 years ago. Since then, the global prevalence of maternal obesity during pregnancy has increased dramatically, to date in some countries exceeding the prevalence observed in our high-risk sample in 2005–2009 [2, 48, 49]. While the risk-factor status limits generalizability from the findings to cohorts enrolled in the same era, they may generalize to cohorts enrolled more recently, in which the prevalence rate of maternal obesity during pregnancy compares with the rate observed in our cohort. Another limitation is that our study may suffer from the selective dropout, as the mothers who provided blood samples during pregnancy and were included in the analytic sample were younger and less likely to be obese than the mothers who did not. However, selective dropout has been found to only marginally alter predictive models [50]. A further limitation is that the study was conducted in high-resource Nordic setting. These limitations restrict generalization from our findings to populations that differ from ours.

In conclusion, our findings suggest that maternal metabolomic perturbations during pregnancy that are specific to her higher early-pregnancy BMI are associated with increased risk of mental and behavioral disorders in children. Our findings may allow identifying metabolomic targets for interventions aimed at promoting mental health in the children of women with higher early-pregnancy BMI.

## Supplementary information

Supplemental Table 1.

Supplemental Table 2.

## Data Availability

Data sets and codes generated during the current study are not publicly available due to protection of personal information but may be made available upon reasonable request. Requests are subject to further review by the national register authority and by the ethical committees. Requests should be made to the PI of the study Professor Katri Raikkonen (katri.raikkonen@helsinki.fi).
