# Supplementary material for: Maternal early-pregnancy body mass index-associated metabolomic component and mental and behavioral disorders in children
Source: Mol Psychiatry. 2022 Aug 10;27(11):4653–61. doi: 10.1038/s41380-022-01723-3 (PMC9734035; doi:10.1038/s41380-022-01723-3)
Supplement: Supplementary file 2 — Supplemental Table 2. [file 41380_2022_1723_MOESM2_ESM.docx]

| Supplemental Table 2. Associations between maternal early-pregnancy body mass index (BMI)-associated metabolomic component based on mean values of 225 metabolites and mental and behavioral disorders in children. | | | |
| --- | --- | --- | --- |
|  | HR/RR | 95% CI | p |
| **Any mental disorder diagnosis** |  |  |  |
| Model 1 | 1.48 | 1.17, 1.87 | 0.001 |
| Model 2 | 1.37 | 1.06, 1.78 | 0.02 |
| **Total number of mental disorder diagnoses** |  |  |  |
| Model 1 | 1.44 | 1.19, 1.74 | 0.0002 |
| Model 2 | 1.37 | 1.11, 1.68 | 0.003 |
| Model 1 is adjusted for child’s sex and survival age/child’s age. Model 2 is additionally adjusted for maternal age, parity, education and smoking and/or alcohol use during pregnancy. | | | |
